# SWAT 76 evaluation: randomised evaluation of sending pre-notification cards to trial participants before a face-to-face primary outcome measurement to increase attendance

**DOI:** 10.12688/f1000research.50890.1

**Published:** 2021-02-08

**Authors:** Shaun Treweek, Stephanie Gallant, Annie S. Anderson

**Affiliations:** 1 Health Services Research Unit, University of Aberdeen, Aberdeen, UK; 2 Division of Molecular and Clinical Medicine, University of Dundee, Dundee, UK; 3 Centre for Research into Cancer Prevention and Screening, University of Dundee, Dundee, UK

**Keywords:** SWAT, retention, pre-notification cards, randomised trial

## Abstract

**Background:** Retention is considered the second highest trial methods priority in the UK after recruitment.

**Methods:** This Study Within A Trial (SWAT) evaluated whether sending a pre-notification card around one month before a face-to-face primary outcome measurement visit compared to not sending the card increased trial retention. The SWAT was a two-arm, parallel randomised (1:1 allocation ratio), stratified by centre, study. It was embedded within the ActWELL host trial, which evaluated whether women receiving lifestyle change counselling from volunteer coaches improved outcomes including weight and physical activity. The text on the card was not developed using formal behavioural change theory but did target factors thought to influence attendance.

The SWAT primary outcome was the difference in the proportion of participants attending the host trial primary outcome measurement visit. The secondary outcome was the direct cost of sending cards. Host trial participants and research staff at the primary outcome visit were blind to the SWAT. Analysis was intention-to-treat. GRADE was used the assess the certainty of evidence.

**Results:** 558 host trial participants took part in the SWAT and were included in the analysis. Sending a pre-notification card may result in a slight increase in attendance at a face-to-face primary outcome measurement visit: risk difference = 3.3% (95% confidence interval = -3.0% to 9.6%). This is GRADE low certainty evidence. A recording error meant it was unclear whether 17 participants allocated to the card were actually sent one but a sensitivity analysis did not change the overall result or conclusion. The direct cost of producing and sending the cards was £192 GBP (€213 EUR; $260 USD).

**Discussion:** Trialists could consider using pre-notification as they may gain a slight increase in retention to face-to-face trial measurement visits but further evaluations are needed.

## Introduction

Retention is considered the second highest trial methods priority in the UK after recruitment ^1^. A recent UK study found that the median loss-to-follow-up in a sample of 151 trials was 11% ^2^. Reminders are generally an effective way of increasing response rates to questionnaires and there is evidence that pre-notification (contacting participants to say that they will soon be sent a questionnaire) is beneficial, although it is not high certainty evidence ^3^.

There is no clear evidence from the Cochrane systematic review of trial retention interventions that pre-notification is effective for trial retention for face-to-face visits ^4^. However, at the time the review was published, an ongoing Study Within A Trial (SWAT) in a trial involving women aged between 70 and 84 years at high risk of osteoporotic fractures did find that sending a newsletter to participants approximately six weeks before a trial questionnaire increased retention by around 1% ^5^.

### SWAT question

Does sending a pre-notification card around one month before a face-to-face primary outcome measurement visit compared to not sending the card increase trial retention?

## Methods

### SWAT protocol

This SWAT is registered on the SWAT repository as SWAT 76. See: http://www.qub.ac.uk/sites/TheNorthernIrelandNetworkforTrialsMethodologyResearch/FileStore/Filetoupload,864300,en.pdf

### Host trial

This SWAT evaluation was embedded in the ActWELL trial ( ISRCTN11057518) ^6^. ActWELL evaluates whether women who receive two, face-to-face lifestyle change sessions from volunteer coaches followed by up to nine telephone calls over a year, compared to no counselling, improves a range of lifestyle outcomes. The two primary outcomes were weight change and change in physical activity at 12-months. Women were invited to take part in ActWELL when they attended their routine mammography appointment (all women aged 50 - 70 in Scotland receive an offer of mammography every three years) at one of four Scottish National Health Service Breast Screening centres. A total of 560 women were randomised into the ActWELL trial.

### Participants

All host trial participants were eligible.

### Intervention

**Figure 1.**
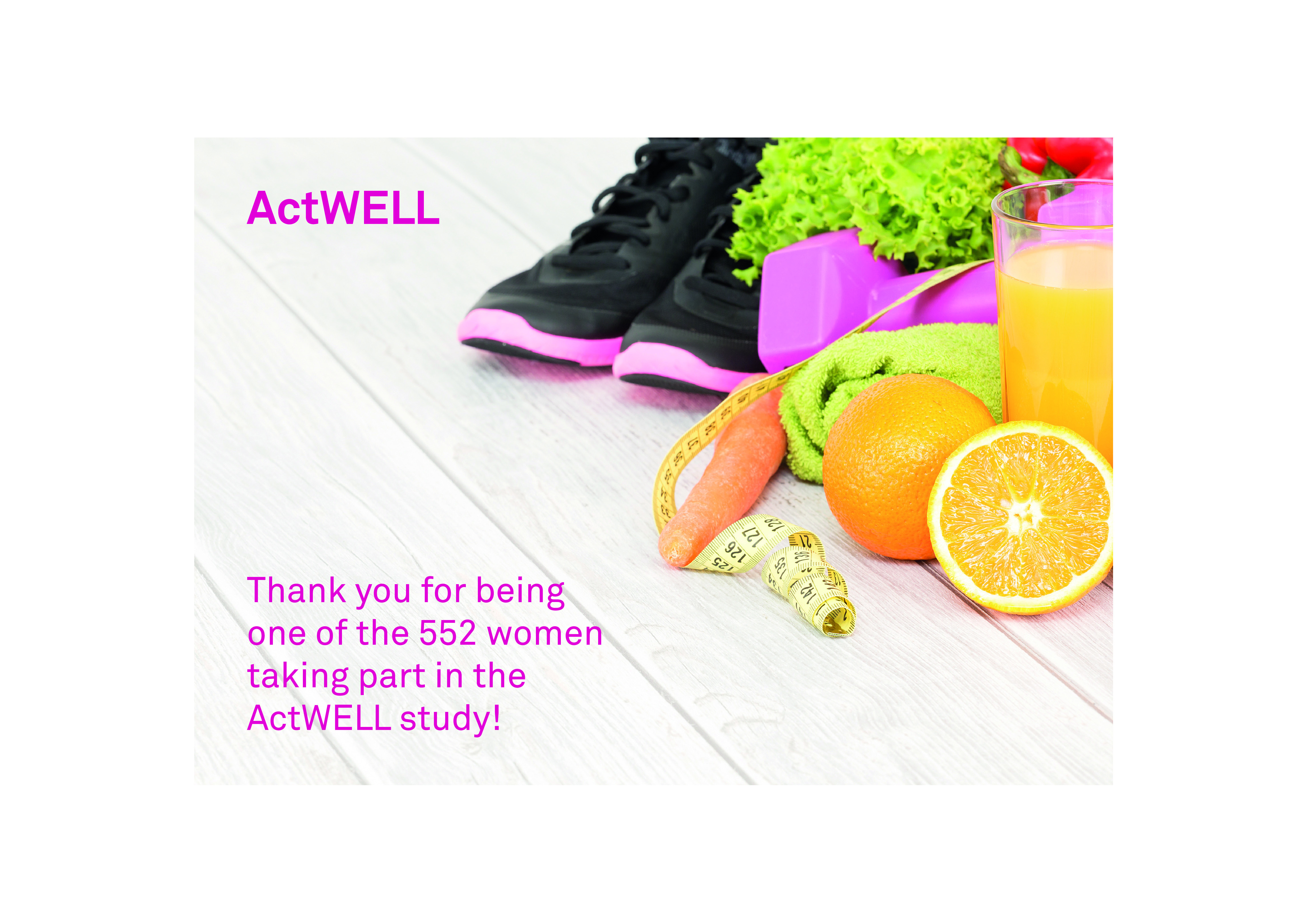

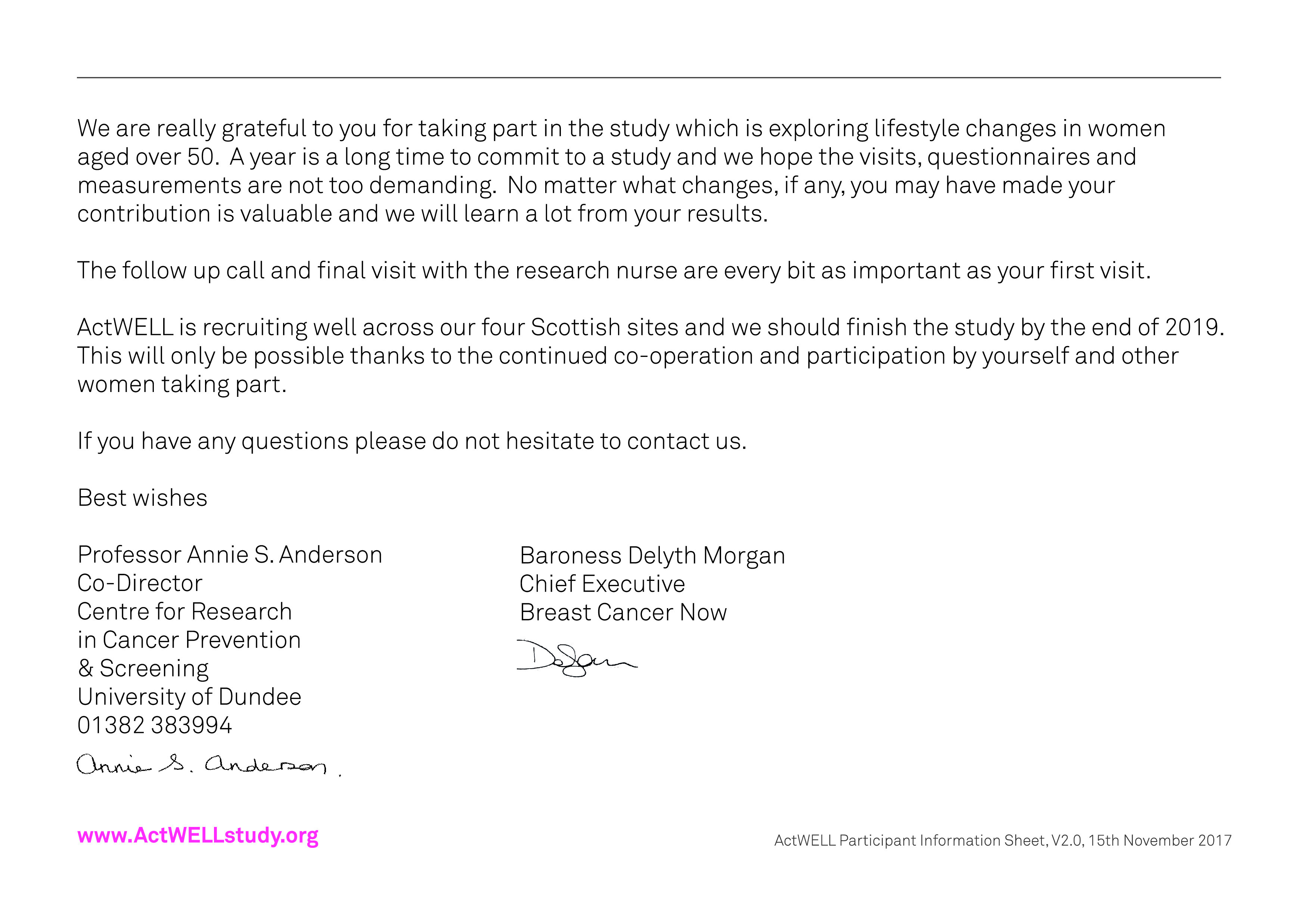
The ActWELL 12-month pre-notification card.

The intervention is a pre-notification card sent around one month before the face-to-face primary outcome measurement visit. The text on the card was not developed using formal behavioural change theory but did target factors thought to influence attendance. Women were thanked to make them feel valued, were told their data were valuable regardless of how things had gone in the trial and the number of other women in the trial was highlighted. The card was signed by the Chief Investigator of the host trial and the Chief Executive of Breast Cancer Now, the charity involved in delivering the host trial intervention. The card is shown in Figure 1.

### Comparator

No pre-notification card.

### Outcomes

*Primary outcome*: the difference in the proportion of participants attending the host trial primary outcome measurement visit (i.e., retention).

*Secondary outcome*: the direct cost of sending pre-notification cards.

### Sample size

The sample size was determined by the host trial so no sample size calculation was done. See Trial Forge Guidance 1 for more information about SWAT sample size calculation ^7^.

### Randomisation

Two-arm, parallel randomised with a 1:1 allocation ratio, stratified by centre. One of the authors (ST) prepared a central randomisation list for each centre for up to 150 participants using https://www.random.org/sequences/. This was then passed to the trial manager and trial administrator who sent out the pre-notification cards.

### Blinding

Women in the host trial had no knowledge of the SWAT. Host trial primary outcome visits were organised and done by research nurses, who had no knowledge of the SWAT or host trial allocation. The SWAT primary outcome, retention, was objective.

### Approvals

The study was approved by East of Scotland Research Ethics Service REC 1 as part of the host ActWELL trial (17/ES/0073). The low risk nature of the SWAT evaluation meant that individual informed consent from host trial participants to be involved was not required by the ethics committee, in line with most SWATs in the UK.

### Statistical analysis

The difference in the proportion of attended visits between groups was calculated using Comprehensive Meta-Analysis Version 3 ( https://www.meta-analysis.com/).

GRADE was used to assess the certainty of the evidence ^8^. In addition to the numerical result, the result is summarised as an informative statement as per GRADE Guidelines 26 ^9^.

## Results

**Figure 2.**
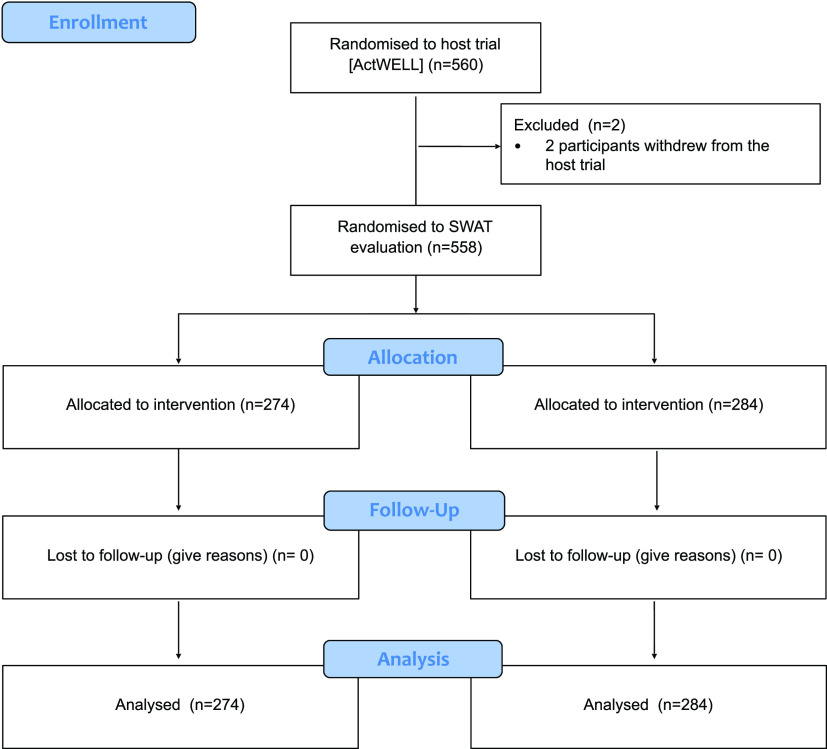
Flow diagram summarising the flow of participants through the SWAT evaluation.

Two host trial participants withdrew before the 12-month host trial primary outcome measurement meaning 558 were included in the SWAT, which ran between March 2018 and July 2019 ( Figure 2; Table 1). One host trial centre recruited 151 participants, which was beyond its recruitment target and one participant beyond the randomisation list for that centre. The extra participant was manually allocated to the comparator arm. A discrepancy between the randomisation log (which indicated who should get a card) and the host trial's tracker system (which confirmed that a card had been sent to a participant) meant that we could not confirm whether 17 participants who should have been sent the pre-notification card were actually sent one. Three further participants who should have received a card are known to have not been sent a card because the participant was called in for a host trial measurement visit before the card could be sent.

The summary statement below and the primary analysis in Table 1 are intention-to-treat as per the randomisation schedule. A sensitivity analysis done using the tracker data is also shown.

**Summary statement of result**: Sending a pre-notification card may result in a slight increase in attendance at a face-to-face primary outcome measurement visit. Risk difference = 3.3% (95% confidence interval = -3.0% to 9.6%). GRADE = low certainty evidence.

**Table 1.** Attendance at the 12-month primary outcome measurement visit for those sent a pre-notification card and those not sent a card. A sensitivity analysis was done to explore the impact of a record-keeping error (see main text).

Attendance at 12-months- Intention-to-treat analysis (SWAT primary analysis)				
Allocated to be sent a pre-notification card (n=274)*		Allocated to not be sent a notification card (n=284)		
Attended visit	Did not attend visit	Attended visit	Did not attend visit	Risk difference (95% confidence interval)
231 (84%)	43 (16%)	230 (81%)	54 (19%)	3.3% (-3.0% to 9.6%)
*This includes three participants who were allocated to receive a card but who are known not to have been sent one.				

**Attendance at 12-months- As per tracker system (sensitivity analysis)**				
**Tracker registers participant was sent a pre-notification card (n=254)**		**Tracker registers participant was not sent a notification card (n=304)**		
Attended visit	Did not attend visit	Attended visit	Did not attend visit	Risk difference (95% confidence interval)
213 (84%)	41 (16%)	248 (82%)	56 (18%)	2.3% (-4.0% to 8.6%)

**GRADE assessment**				
**Study limitations**	No serious issues.			
**Inconsistency of results**	Downgrade 1 level because this is a single study (sparsity of data).			
**Indirectness of evidence**	No serious issues.			
**Imprecision**	Downgrade 1 level because confidence interval is wide and crosses risk difference of 0.			
**Reporting bias**	No serious issues.			

### Direct costs

The direct costs of printing the cards was £72 GBP. Design work was extremely modest, bundled with other host trial design work and not charged separately. Second class (i.e., delivery within two days) postage costs were run through the University of Dundee mailroom at an estimated cost of £120 GBP. The total direct cost was therefore £192 GBP (€212 EUR; $259 USD).

## Discussion

Sending a simple card about one month prior to a face-to-face primary outcome measurement visit may result in a slight improvement in attendance. This is GRADE low certainty evidence because there is just this single evaluation and it is imprecise.

We are not aware of other pre-notification interventions that target face-to-face trial visits. An upcoming 2021 update of the Cochrane retention review ^4^ found no additional pre-notification studies (ST is a co-author of this update). Mitchell and colleagues ^5^ added their evaluation to a meta-analysis of pre-notification evidence done outside trials and healthcare. As might be expected, there was substantial heterogeneity but the overall direction of effect was also in favour of pre-notification.

### Strengths and limitations

We had a record-keeping error, which means we cannot say with confidence that all participants who should have been sent a pre-notification card were sent one. However, the number of participants affected was relatively small and our overall results and conclusion remain the same regardless of whether we analyse according to the randomisation schedule or the tracker system. There are currently only two evaluations of pre-notification in trials, our own of cards aimed at face-to-face visits and that of Mitchell and colleagues of a newsletter to increase questionnaire response ^5^. This does not provide a broad range of contexts for this evidence base. Both evaluations were done in the UK and in women only.

### Implications for trial practice

Trialists could consider using pre-notification as they may gain a slight increase in retention to face-to-face trial measurement visits.

### Implications for SWAT research

Looking at the existing evidence and referring to Trial Forge Guidance 2 as to whether further SWATs evaluating this intervention are required ^10^, we conclude that more evaluations are needed because the GRADE certainty in the evidence is not high, there is only a single evaluation meaning cumulative meta-analysis cannot converge and few host trial contexts are included.

Further evaluations of pre-notification in trials could target either face-to-face or questionnaires but should aim to add new host trial contexts. Future host trials should involve men. Formal approaches to developing intervention content may increase effect sizes.

## Data availability

### Underlying data

Open Science Framework: SWAT 76 data for host trial ActWELL, https://doi.org/10.17605/OSF.IO/N64HU ^11^

This project contains the following underlying data:

- Primary analysis 8-1-2021 (public).csv
- Sensitivity analysis 8-1-2021 (public).csv

### Reporting guidelines

Open Science Framework: CONSORT checklist for ‘SWAT 76 evaluation: randomised evaluation of sending pre-notification cards to trial participants before a face-to-face primary outcome measurement to increase attendance’, https://doi.org/10.17605/OSF.IO/B78JT ^12^

Data are available under the terms of the Creative Commons Attribution 4.0 International license (CC-BY 4.0).
