# Supplementary material for: SWAT 76 evaluation: randomised evaluation of sending pre-notification cards to trial participants before a face-to-face primary outcome measurement to increase attendance
Source: F1000Res. 2021 Jul 15;10:84. Originally published 2021 Feb 8. [Version 2] doi: 10.12688/f1000research.50890.2 (PMC8080942; doi:10.12688/f1000research.50890.2)
Supplement: Peer review history

# Peer review: Reviewer response for version 2

**DOI:** 10.5256/f1000research.58441.r89686

**Published:** 2021-08-11

**Authors:** Christopher Sutton

**Affiliations:** 1 Centre for Biostatistics, University of Manchester, Manchester, UK

I am pleased to have had the opportunity to review and comment on the revised article and can confirm that I support the decisions and amendments made by the authors and have no further comments.

Is the work clearly and accurately presented and does it cite the current literature?

Yes

If applicable, is the statistical analysis and its interpretation appropriate?

Partly

Are all the source data underlying the results available to ensure full reproducibility?

Yes

Is the study design appropriate and is the work technically sound?

Yes

Are the conclusions drawn adequately supported by the results?

Yes

Are sufficient details of methods and analysis provided to allow replication by others?

Yes

Reviewer Expertise:

Clinical trial methodology, particularly around trial conduct and retention methods.

I confirm that I have read this submission and believe that I have an appropriate level of expertise to confirm that it is of an acceptable scientific standard.

# Peer review: Reviewer response for version 1

**DOI:** 10.5256/f1000research.53982.r79341

**Published:** 2021-04-27

**Authors:** Michelle E. Kho

**Affiliations:** 1 School of Rehabilitation Science, McMaster University, Hamilton, ON, Canada

Treweek *et al.* conducted a study within a trial to evaluate whether a post card sent 1 month prior to an in-person primary outcome research visit improved retention within the multicentre ActWELL randomized trial of lifestyle coaching. 558 participants from the host trial were sent a pre-notification card. Of this cohort, 84% who were allocated a pre-notification card attended the primary outcome visit, compared to 81% who were not allocated a pre-notification card. This is a well-written, well-designed, and well-reported study. It will make important contributions to further understanding ways to improve retention to clinical trials. I have some considerations for the authors.

**Moderate issues:**

1. Results – To help the reader understand the impact on the trial, I recommend the authors first report the total number of women who attended their primary outcome follow-up visit, then report the proportion by group.
2. Results – I initially found Table 1 confusing. Since those who did not attend the visit is the complement of those who did attend a visit, I suggest reporting only those who attended the visit. The risk difference represents the difference between those who attended the visit. I also suggest adding the 95% CI around the estimates, and report 1 decimal point for consistency with the risk difference.
3. Can the authors report whether patients recalled receiving the pre-notification card before their outcomes visit?
4. Implications for trial practice – I suggest adding text to further contextualize the implications of the relative cost of this intervention, the overall investment costs in the enrolled patients to-date, and the importance of ascertaining the primary outcome. Even a 3% improvement in primary outcome ascertainment could affect trial results.

**Minor issues:**

1. Abstract – I suggest reporting absolute values in the intervention and control groups, then the risk difference.
2. Introduction – I suggest adding a statement regarding the importance of the primary outcome in a trial and the need for interventions to optimize ascertainment.
3. Intervention – I suggest moving the text regarding behavioural change theory in the development of the pre-notification card from the methods to the limitations section of the manuscript.
4. Results – Data error for 17 participants (3%) – suggest also reporting % of total group to help the reader easily evaluate the small number. Interestingly, the risk difference was also 3%. The authors conducted a sensitivity analysis, and the results were similar. I suggest this is also a strength of the study.
5. Implications for trial practice. I suggest framing the study findings specific to 1-year follow-up measures.

Is the work clearly and accurately presented and does it cite the current literature?

Yes

If applicable, is the statistical analysis and its interpretation appropriate?

Partly

Are all the source data underlying the results available to ensure full reproducibility?

Yes

Is the study design appropriate and is the work technically sound?

Yes

Are the conclusions drawn adequately supported by the results?

Yes

Are sufficient details of methods and analysis provided to allow replication by others?

Yes

Reviewer Expertise:

Critical care clinical trials; health research methodology; rehabilitation; physiotherapy

I confirm that I have read this submission and believe that I have an appropriate level of expertise to confirm that it is of an acceptable scientific standard.

# Response

**Published:** 2021-07-05

**Authors:** Shaun P. Treweek

**Affiliations:** University of Aberdeen, UK

**Response to comments on F1KR00CDE F1R-VER53982-A**

**5/7/2021**

 Thanks for the reviewers’ comments on our manuscript. Our responses are below.  We have made the necessary changes to the manuscript using track changes so that they can be easily seen. We have also submitted a clean version with the changes accepted.

**Reviewer #1**

*1. Introduction:  clearly state that the previous SWAT of the impact of a newsletter on retention was statistically significant (p<0.05 … just!)*

 Done. We have also added the exact difference of 1.6% rather than saying ‘around 1%’.

*2. I would query the statement that “All trial participants were eligible.”*

 We have changed our text to:

 ‘All host trial participants who had not withdrawn were eligible.’

*3. The primary outcome would be better specified as a binary outcome measure (i.e. at the participant level e.g. “attendance at the host trial primary outcome measurement visit”).*

 We have thought about this but since the primary result we report is the difference in attendance between those who got the card and those who did not (i.e. 3.3%) and not the actual attendance for each group (84% and 81%), we think the way we describe the primary is correct.

*4. Statistical analysis: the method of analysis should be stated, in addition to the software used*

 We have added that we used a fixed effects model.

*5. Present relative relative measures of effect as well.*

 Good idea, apologies for not doing it originally. Done.

*6. I would like to see more information on the economic analysis.  For example, how much did the intervention cost per participant retained?*

 We only collected direct costs so our economic analysis is very limited. However, we liked the suggestion to calculate a cost per (extra) participant retained, which means we have added the text below to the cost information. We have also added the cost per additional participant retained to the abstract.

 ‘ **Cost per additional participant retained**

 If the 274 participants who received the card had attended at the same rate as those who did not, a total of 222 participants would have attended the next visit. In fact, 231 attended, meaning an extra nine participants were retained. The cost per additional participant retained was £192/9, or £21.33 (€23.55; $28.77).’

*7. In the Discussion and Strengths and Limitations, the authors state that there are no additional pre-notification studies; it would be better to stated that there are no additional completed pre-notification studies*

 We have now amended the Discussion text to:

 ‘The 2021 update of the Cochrane retention review [4] found no additional completed pre-notification studies (ST is a co-author of this update). There are at least two pre-notification protocols (SWAT 77 and SWAT 86) registered on the SWAT repository (https://www.qub.ac.uk/sites/TheNorthernIrelandNetworkforTrialsMethodologyResearch/SWATSWARInformation/Repositories/SWATStore/) in addition to our own SWAT 76, meaning there may be additional studies underway but not yet complete.’

*8. Reference list: references 11 and 12 re identical (Reference 12 should refer to the CONSORT checklist for this SWAT).*

 References 11 and 12 were added by the publisher, not us, but they do work as they should in the version I downloaded yesterday (8/6/2021). There may perhaps have been an error in the version the reviewer was sent that has now been corrected by the publisher.

**Reviewer #2**

*1. Results – To help the reader understand the impact on the trial, I recommend the authors first report the total number of women who attended their primary outcome follow-up visit, then report the proportion by group.*

 We have added this number to the header row of Table 1 and we have done this for both the intention-to-treat and sensitivity analyses.

*2. Results – I initially found Table 1 confusing. Since those who did not attend the visit is the complement of those who did attend a visit, I suggest reporting only those who attended the visit. The risk difference represents the difference between those who attended the visit. I also suggest adding the 95% CI around the estimates, and report 1 decimal point for consistency with the risk difference.*

 We have thought about the reviewer’s comment re. only showing the number who attended a visit but have decided that we would prefer to be explicit and give both the number who did attend and the number who did not. The main reason is that it avoids the reader having to do a calculation and all the raw numbers are there for readers to do other calculations should they wish.

 There is no confidence interval for the proportions attending a visit: the numbers are not an estimate but the actual number attending. The risk difference (and now relative risk in response to Reviewer 1, Comment #5) does have a confidence interval around it. We have  changed our presentation so that we now consistently report to 1 decimal place as requested by the reviewer.

*3. Can the authors report whether patients recalled receiving the pre-notification card before their outcomes visit?*

 Participants were not asked whether they recalled getting a card so we cannot unfortunately report on this. Both the participants and the research nurses who took measurements at the primary outcome visits did not know that we were running this study (i.e. they were blind) and to maintain the blind, we did not ask about the cards. It is possible that some participants mentioned this to the nurse but we have no record of this.

*4. Implications for trial practice – I suggest adding text to further contextualize the implications of the relative cost of this intervention, the overall investment costs in the enrolled patients to-date, and the importance of ascertaining the primary outcome. Even a 3% improvement in primary outcome ascertainment could affect trial results.*

 Good point.  We have re-written the *Implications for trial practice* section:

 ‘Given the paucity of evidence to support retention decisions [Reference 4], trialists could consider using pre-notification as they may gain a slight increase in retention to face-to-face trial measurement visits. Trials are expensive and in that context pre-notification cards are a very cheap intervention that may provide a small increase in the proportion of primary outcome data a trial team obtains. We had no negative reaction to them from participants (i.e. there were no complaints) and our cost of around £21 per additional retained participant is likely to be substantially cheaper that recruiting an additional participant to replace these lost primary outcome data.’      

    

*5. Abstract – I suggest reporting absolute values in the intervention and control groups, then the risk difference.*

 Done.

*6. Introduction – I suggest adding a statement regarding the importance of the primary outcome in a trial and the need for interventions to optimize ascertainment.*

 We have added the following text to the Introduction:

 ‘This reduces the amount of trial data available for analysis, which is especially problematic for the trial’s most important outcome– the primary outcome– because this is the outcome that will be used to judge whether the trial intervention is effective. Ensuring that retention is high is therefore of great importance to trialists.’

*7. Intervention – I suggest moving the text regarding behavioural change theory in the development of the pre-notification card from the methods to the limitations section of the manuscript.*

 Done.

*8. Results – Data error for 17 participants (3%) – suggest also reporting % of total group to help the reader easily evaluate the small number. Interestingly, the risk difference was also 3%. The authors conducted a sensitivity analysis, and the results were similar. I suggest this is also a strength of the study.*

 We have added the proportion (3%) to the Results and also mentioned that the similarity of the sensitivity analysis results to the intention-to-treat results shows the results are robust in the face of this recording error.   

*9. Implications for trial practice. I suggest framing the study findings specific to 1-year follow-up measures.*

 We have made the change suggested and also added ‘at 1-year’ to our evidence summary statement in the Abstract and Results.

# Peer review: Reviewer response for version 1

**DOI:** 10.5256/f1000research.53982.r79342

**Published:** 2021-03-01

**Authors:** Christopher Sutton

**Affiliations:** 1 Centre for Biostatistics, University of Manchester, Manchester, UK

Overall, this is an excellent manuscript and an extremely welcome and useful addition to the literature on trial retention.  The work provides a clear citation of the relevant literature.

 The methodology is sound and the trial performed well; it has significant academic merit. There are, however, aspects of the methods and analysis which are a little under-specified and unclear, and suggestions for clarification are detailed below.  Likewise, the statistical and economic analysis is appropriate, although I do suggest additional analyses and results be presented to enhance interpretation and generalisability. 

 The source data underlying the results are available.  The conclusions are drawn adequately and supported by the results, with an appropriate sensitivity analysis performed to investigate the potential impact of some degree of failure to definitively send the pre-notification card to 20 participants allocated to receive the card.

 Specific comments:

1. Introduction:  clearly state that the previous SWAT of the impact of a newsletter on retention was statistically significant (p<0.05 … just!).
2. I would query the statement that “All trial participants were eligible.”  From my perspective, the eligibility criteria were (a) Trial participant of the ActWELL trial (b) remaining in ActWELL at 1 month prior to the 12-month final follow-up.
3. The primary outcome would be better specified as a binary outcome measure (i.e. at the participant level e.g. “attendance at the host trial primary outcome measurement visit”).
4. Statistical analysis: the method of analysis should be stated, in addition to the software used.
5. In line with CONSORT 2010, for binary outcome measures both absolute and relative measures of effect are recommended.  Across different trial populations, interventions, duration of follow-up etc., it is likely that there are different ‘control’ rates of retention and the impact of any intervention is probably more likely to be on a relative rather than an absolute scale.  For example, the newsletter trial referenced in the Introduction (Mitchell *et al*., (2012)) had a control group retention rate of 94.6% (and an absolute increase of 1.6% in the intervention group), compared with this trial which had a control group retention rate of 81.0% (and an absolute increase of 3.3%).  It is therefore recommended that the results are also presented as a Risk Ratio (Relative Risk) or Odds Ratio.
6. I would like to see more information on the economic analysis.  For example, how much did the intervention cost per participant retained?
7. In the Discussion and Strengths and Limitations, the authors state that there are no additional pre-notification studies; it would be better to stated that there are no additional *completed* pre-notification studies (there are at least two other pre-notification trials registered on the SWAT Repository Store (SWAT77 and SWAT86).
8. Reference list: references 11 and 12 re identical (Reference 12 should refer to the CONSORT checklist for this SWAT).

Is the work clearly and accurately presented and does it cite the current literature?

Yes

If applicable, is the statistical analysis and its interpretation appropriate?

Partly

Are all the source data underlying the results available to ensure full reproducibility?

Yes

Is the study design appropriate and is the work technically sound?

Yes

Are the conclusions drawn adequately supported by the results?

Yes

Are sufficient details of methods and analysis provided to allow replication by others?

Yes

Reviewer Expertise:

Clinical trial methodology, particularly around trial conduct and retention methods.

I confirm that I have read this submission and believe that I have an appropriate level of expertise to confirm that it is of an acceptable scientific standard.
